# Anaemia in HIV-infected children: severity, types and effect on response to HAART

**DOI:** 10.1186/1471-2431-12-170

**Published:** 2012-10-31

**Authors:** Eunice Nyesigire Ruhinda, Francis Bajunirwe, Julius Kiwanuka

**Affiliations:** 1 Department of Paediatrics and Child Health-Mbarara, University of Science and Technology, P.O. BOX 1410, Mbarara, Uganda; 2 Department of Community Health-Mbarara, University of Science and Technology, P.O. BOX 1410, Mbarara, Uganda; 3 Department of Paediatrics and Child Health-Mbarara, University of Science and Technology, P.O. BOX 1410, Mbarara, Uganda

## Abstract

**Background:**

HIV and anaemia are major health challenges in Africa. Anaemia in HIV-infected individuals is associated with more rapid disease progression and a poorer prognosis if not addressed appropriately. This study aimed at determining the severity and types of anaemia among HIV infected children and its effect on short term response to antiretroviral therapy (ART).

**Methods:**

At baseline, clinical and haematological parameters of 257 HIV-infected ART-naïve children aged 3 months to 18 years were assessed to determine the prevalence, severity and types of anaemia. ART eligible patients were started on therapy according to WHO criteria, enrolled (n=88) into an observational cohort and followed up for 6 months.

**Results:**

Anaemia was present in 148/257 (57.6%) of children, including (93/148) 62.2% with mild anaemia, 47/148 (32.0%) moderate anaemia, and 7/148 (4.8%) with severe anaemia. The mean haemoglobin (hb) was lower among children with more advanced HIV disease (p<0.0001). Microcytic-hypochromic anaemia (44.9%) was the commonest type of anaemia. Anaemia was independently associated with young age (p <0.0001), advanced HIV WHO disease stage (p = 0.034) and low CD4 percentage (p = 0.048). The proportion of children who had attained viral suppression (viral load <400 copies/ml) at 3 months was significantly lower among the anaemic children, 31/58 (53.4%) compared to the non-anaemic children 26/30 (86.7%) (p=0.002). However, the difference in clinical and immunological response between the anaemic and non-anaemic patients did not reach statistical significance.

**Conclusion:**

Anaemia is highly prevalent among HIV-infected children in a rural Ugandan clinic and is associated with poorer virological suppression. However, the anaemia did not impact clinical and immunological response to ART among these children.

## Background

Anaemia has been recognized as an important clinical problem in HIV-infected patients [1-3] with an estimated prevalence ranging from 10% in asymptomatic HIV-infected patients to 92% in patients with AIDS [4,5]. In Uganda, anaemia was shown to have a high prevalence of 92% and an overall cumulative incidence of 100% among young HIV-infected children at baseline in a hospital-based cohort [6].

Studies done earlier found an association between the presence of anaemia at baseline and decreased survival as well as increased disease progression in patients with HIV infection [7,8]. Even with antiretroviral therapy (ART), anaemia remains strongly and consistently associated with HIV disease progression [8], and reversal of anaemia in HIV was shown to be associated with increased life expectancy in adults [9,10].

Most of the studies on the HIV-anaemia interplay were done in developed countries and mainly included adults. The effect of the presence of anaemia on response to ART in HIV-infected children has not been studied. This study described the prevalence, severity and types of anaemia among ART-naïve HIV-infected children presenting at Mbarara Regional Referral Hospital (MRRH), a tertiary centre in western Uganda, and examined the effect of baseline anaemia on subsequent response to ART.

## Methods

### Design

We used a hybrid of two designs. First, we conducted a baseline evaluation of newly diagnosed HIV positive ART-naïve patients aged 3 months to 18 years attending MRRH from November 2007 to June 2009. HIV-1 is the vastly predominant strain in this region of Africa [11]. The disease stage, prevalence and types of anaemia were described. Second, the children eligible for initiation of ART from the cross-sectional evaluation were consecutively enrolled into a prospective observational HIV treatment cohort and followed for 6 months to measure response to antiretroviral therapy using viral load suppression and immunological markers.

### Laboratory assessment

All patients were subjected to a standardized laboratory assessment consisting of haematological, virological and clinical chemistry tests prior to initiation and during follow-up on ART. Haematological indices including, haemoglobin concentration (Hb), red blood cell indices (mean corpuscular volume [MCV] and mean corpuscular haemoglobin [MCH]), were determined using an automated hematology analyser - COULTER® Ac·T™ 5diff CP (Cap Pierce) [12]. A thin blood film was done and classified either as normocytic-normochromic, hypochromic-microcytic hyperchromic-macrocytic or mixed picture. Reticulocyte counts were determined using the supravital staining method [13]. A reticulocyte count above 2.5% was considered high [14].

CD4/CD8 counts were measured by Flowcytometry using a BD FACSCalibur™ flow cytometer [15]. Viral load (quantitative HIV- RNA PCR) was determined by the standard method using Cobas® Amplicor Analyzer [16].

### Treatment

Eligibility for initiation of ART was based on clinical and immunological criteria outlined in the then current WHO guidelines [17]. The first-line ART regimen comprised of two nucleoside reverse transcriptase inhibitors (zidovudine and lamivudine), and one non-nucleoside reverse transcriptase inhibitor (nevirapine). Efavirenz replaced nevirapine if a child was on concurrent treatment for tuberculosis with rifampicin. Stavudine replaced zidovudine if a child had an Hb <8 g/dl.

### Outcome measures

Anaemia was defined and graded according to age-appropriate reference standards published by WHO [18].

Patients started on ART were reassessed for short-term outcome at 3 and 6 months. Their clinical status was assessed on occurrence of opportunistic infections (O.Is) and growth indices. Immunological response was measured by increase in CD4+ from baseline; defined as adequate if increase was ≥ 10% at 3 months, and ≥ 15% at 6 months. Virological response was measured as a fall in viral load from baseline; defined as adequate if viral load was <400 copies/ml by 6 months.

### Analysis

Sample size calculation was aimed at detecting a difference in the mean rise in CD4% on ART between anaemic and non-anaemic children at baseline. A sample size of 128 patients (64 per group) gave a power of 80% to detect a difference of 7 percentage points with a two sided alpha type 1 error of 0.05. Data were analyzed using SPSS® program version 12.0.

Contingency tables were constructed to determine the relationship between anaemia and HIV disease stage. Odds ratios with 95% confidence intervals were calculated to determine the strength of association between the presence of anaemia and the HIV disease stage. For categorical variables with more than two categories such as types of anaemia, Chi-Square test for association was used to determine the association. Significant factors were included in a multiple logistic regression model to determine those which were independently associated with presence of anaemia.

To estimate the impact of baseline anaemia on short-term response to ART, comparisons of immunological and virological responses between anaemic and non-anaemic groups were conducted using two methods. First, the proportion of children who registered a rise in CD4% of 10% at 3 months and 15% at 6 months was compared between the two categories (anaemic vs. non-anaemic) for children less than 5 years [for children above 5 years an equivalent response was defined as a rise of 50% on the baseline absolute count at 3 months, and doubling the baseline CD4+ count by 6 months]. Secondly, a comparison of mean rise in CD4% at 3 and 6 months was done between initially anaemic and non-anaemic children.

To assess viral load response, we calculated the proportion of children who had achieved viral load suppression to undetectable levels at 3 and 6 months of ART by anaemia status. Means of viral load were also calculated for the two groups and compared at different follow-up time points using the t- test. These variables; CD4+ and viral load were tested and met the assumptions for the t-test.

### Ethical considerations

The research was approved by the Faculty of Medicine Research and Ethics Committee and the Institutional Review Board of Mbarara University. All mothers/guardians of enrolled children gave written informed consent to participate in the study. Patients received the standard of care, including free ART and management of anaemia according to standard protocol.

## Result

### Baseline assessment

A total of 276 HIV-infected children presented to the HIV clinic at MRRH between November 2007 and June 2009, but only 257 patients met the criteria and were enrolled into the study. We excluded 19 children who were already receiving ART. Of the 257 recruited patients, 23(8.9%) were in WHO clinical stage I, 66 (25.7%) were in stage II. During the analysis, stage I and II were combined and termed as early stage disease. WHO clinical stage III had 117(45.5%) while 51(19.8%) were in stage IV (AIDS). Stage III and IV were also grouped together and called late HIV clinical stage and therefore met the WHO criteria to start ART. They accounted for 168 (65.4%) of the total patients. However, only 98 (59%) were started on ART in time for the study. The age range was 4–207 months with a median of 48 months (SD 54.6), but 224(87%) were 12–180 months.

Haemoglobin levels ranged from 4.3-16.0g/dl with a median Hb of 10.5g/dl (SD 2.01). A total of 148 out of 257 (57.6%) patients had anaemia at enrolment. Most patients (62.2%) had mild anaemia, 32.0% had moderate anaemia while 4.8% had severe anaemia. The most common type of anaemia was microcytic-hypochromic in 66 (44.9%) patients, followed by normocytic-hypochromic in 39 (26.5%), 28 (19.0%) patients had normocytic-normochromic anaemia and 12 (8.2%) patients had a mixed picture.

### Comparison of anaemia and no anaemia groups

The younger the patients, the more likely they were to be anaemic (p< 0.0001). Other factors significantly associated with the presence of anaemia included presence of OIs, previous hospital admission, and presence of severe malnutrition (weight for age Z-scores (WAZ) or weight for height Z-scores (WHZ) <−3.0 SD). The use of co-trimoxazole (TMP/SMZ) for *Pneumocystis jiroveci* pneumonia (PJP) prophylaxis was also significantly associated with anaemia (Table 1).

**Table 1:** Patient characteristics at recruitment

**Characteristic**	**Anaemia**	**No anaemia**	**Chi**-**square**	**P value**
	**n = 148 (%)**	**n = 109 (%)**		
**Gender**				
Female	85 (57.4)	53 (48.6)	1.959	0.167
**Residence**				
Rural	110 (74.3)	82 (75.2)	0.027	0.886
**Age categories**				
< 12 months	22 (14.9)	3 (2.8)		
12-59 months	89 (60.1)	31 (28.4)		
60 months and above	37 (25)	75 (68.8)	50.613	<0.0001*
**Previous admission**	113 (76.4)	48 (44)	28.01	<0.0001*
**Presence of OI**	108 (73.0)	58 (53.7)	10.170	0.001*
**Nutritional status**				
Mean WAZ	−2.46	−1.66		<0.0001*
Mean WHZ	−2.54	−2.08		0.023*
MUAC for the < 5 (mean)	11.99	13.54		0.001*
**Co-trimoxazole use**	50 (33.8)	20 (18.3)	7.546	0.004*
**Baseline reticulocyte count**	3.1	1.2		<0.0001*

WAZ = Weight for age Z scores. WHZ = Weight for height Z scores. MUAC = Mid upper arm circumference. * Statistically significant.

Reticulocyte counts ranged from 0.3-11.6. Anaemic patients had a significantly higher mean reticulocyte count of 3.1 compared to 1.2 (p <0.0001) among non anaemic patients.

HIV disease stage (clinical and immunological) was significantly associated with both the presence and severity of anaemia. Late stage HIV-disease (WHO stage III and IV) patients were 4 times more likely to be anaemic. Mean CD4+ values were significantly lower in the anaemic group (p = 0.04) (Table 2).

**Table 2:** Anaemia and clinical/immunological stage

**Disease stage at baseline**	**Anaemia**	**No anaemia**	**P value**
	**n = 148 (%)**	**n = 109 (%)**	
**Clinical disease stage**			
Early (Stage I &II)	31 (20.9)	58 (53.2%)	<0.0001
Late (Stage III &IV)	117 (79.1)	51 (46.8%)	
**Immunological disease stage**			
No or mild ISS^§^	65 (43.9)	70 (64.2%)	0.001*
Advanced or Severe ISS^§^	83 (56.1)	39 (35.8%)	
**Mean CD4 percentage**			
**Age** under 5 years	24.74 (n=111)	32.32 (n=34)	0.011*
Age over 5 years	18.11 (n=37)	27.48 (n=75)	0.002*
Combined mean CD4+ %	23.27	28.71	0.004*
**Mean CD4 absolute count**			
Over 5 years	257.32	489.01	<0.0001*

* indicates a significant p value.

^§^ ISS=Immune Suppression Syndrome.

The severity of anaemia generally increased with advancing WHO clinical stage (p<0.0001). Forty four out of 47 (96%) patients with moderate anaemia and all 7 patients with severe anaemia were in stage III or IV. Anaemic patients had a significantly higher baseline viral load (5.23 log _10_) compared to 4.92 log _10_ for the non-anaemic group (p = 0.011).

A logistic regression analysis showed age, CD4+ percentage and WHO clinical staging to be independently associated with the presence of anaemia.

### Anaemia and response to HAART

Anaemic patients had a mean CD4% rise of 8.8 compared to 11.9 for the non anaemic patients at 3 months (p= 0.38). At 6 months, the anaemic patients had attained a mean CD4+ rise of 14.9 compared to the non-anaemic with 15.9. This difference was not significant (p=0.751).

### Viral load response

The mean fall in viral load at 3 months was significantly lower (1.92 log_10_) among anaemic patients compared to 2.42 log_10_ for non-anaemic patients (p = 0.002). Overall, the proportion of children who attained a viral load suppression to undetectable levels by 3 months was higher among non-anaemic compared to the anaemic children (p=0.002) (Table 3).

**Table 3:** Viral load response

**Time**	**Mean viral load**			**Mean fall in viral load**			**% with viral load < 400**		
	**A +**	**A-**	**p value**	**A+**	**A-**	**p value**	**A+ n (%)**	**A- n (%)**	**p value**
**Baseline**	5.23	4.94	0.011^§^	--	--		--	--	
**3 months**	3.40	2.85	0.006^§^	1.92	2.42	0.020^§^	31/58 (53.4)	26/30 (86.7)	0.002^§^
**6 months**	3.44	2.97	0.028^§^	1.92	2.33	0.098	26/48 (54.2)	24/31 (77.4)	0.055

A+ =anaemia A- = no anaemia.

^§^Statistically significant.

## Discussion

Our study found a high prevalence of anaemia among HIV-infected paediatric patients presenting at an urban HIV clinic in Uganda, majority of them with mild anaemia. The frequency as well as severity of anaemia correlated with advanced clinical and immunological HIV disease stage. The study also found a significant association between the presence of anaemia and a poorer short-term virologic response to HAART.

Anaemia in HIV-infected children is multi-factorial and takes varying morphological types [19,20]. As in several other similar studies, we found microcytic-hypochromic anaemia to be the commonest type, especially in advanced HIV disease stages. Although this study did not do specific tests for iron status, it can be expected that iron depletion could have contributed to the high proportion of microcytic-hypochromic anaemia. Alternatively, anaemia of chronic disease – in this case due to advanced HIV disease and related co-morbidities - has also been known to present a similar morphological picture. Another well studied cause of anemia in adults is the presence of antibodies to endogenous erythropoietin [21]. This in children has not been studied.

Although the mean reticulocyte count was significantly higher among patients who were anaemic at baseline, about 25% of these patients had a normal or low reticulocyte count at baseline suggesting either bone marrow suppression as the cause or, at least, a sub-optimal bone marrow response to the anaemia.

In this study, advanced HIV clinical stage [22] was found to be independently associated with the presence of anaemia at baseline. In fact, almost all anaemic patients in early HIV disease stages had mild anaemia; moderate and severe anaemia was found exclusively among patients in stages III and IV. Similarly, the CD4% was significantly associated with the likelihood of anaemia, even after controlling for WHO clinical stage. This association between anaemia and advancing HIV disease has been reported previously [7,9,23] and could be explained by the increasing viral burden which may cause anaemia through increased cytokine-mediated myelosuppression, and/or a higher burden of co-morbidities.

As would be expected from previous studies [24,25] most patients showed significant clinical, immunological and virological response to ART, including those with advanced or severe HIV disease. Notably, a significant proportion of children with anaemia at baseline showed improvement in haemoglobin and related haematological indices while on ART, even without specific additional treatment targeting anaemia. This observation has been reported previously and reinforces the suggestion that a proportion of these cases of anaemia were related directly to chronic HIV infection [26].

Virological response, however, was significantly better among non-anaemic patients compared to those with anaemia, especially in the first 3 months on ART. Compared to initially anaemic patients, non-anaemic patients achieved a higher mean reduction in viral load and a significantly higher proportion attained complete viral suppression. Several previous studies have highlighted the importance of anaemia as an independent prognostic factor in HIV-infected individuals. Anaemia increases the risk of progression to AIDS [8-10] as well as the risk of death in HIV infected patients, both in cross-sectional [10] and longitudinal studies [8,9,23]. The adverse long term outcomes more likely reflect anaemia as a proxy of more advanced disease.

In this study we focused on the more immediate outcomes of treatment with antiretroviral drugs, i.e. viral suppression, rise in CD4+ counts and short-term clinical improvement. Our findings, while reaffirming the importance of anaemia as a poor prognostic factor in HIV-infected children, suggest that the observed poorer outcome in anaemic HIV-infected children is, at least in part, mediated by a suboptimal virological response to HAART. The mechanism of the blunted virological response observed in anaemic children is not clear. Our study enrolled only ART-naïve children and did not include testing for viral resistance to antiretroviral drugs. It is possible that anaemic children who did not respond adequately to ART harboured some drug resistant strains; the risk of this could be expected to be higher in relation to the higher baseline viral loads observed among anaemic children. Other mechanisms might relate to possible poorer bioavailability of drugs in the sicker, anaemic children.

Regardless of the mechanism, recognition of anaemia at initiation of ART could be useful to alert clinicians to children requiring closer monitoring for possible treatment failure. Furthermore, since correction of anaemia has been shown to improve prognosis, monitoring of haemoglobin during ART should help identify those children whose anaemia does not quickly respond to ART and thus require further investigation and specific treatment to correct anaemia.

Results of this study need to be interpreted cautiously in light of some important limitations. Firstly, the exact causes of anaemia in these patients were not determined. A causal relationship should not be inferred about the association between anaemia and ART response, however using our multiple regression analysis, anemia remained a significant predictor of virological response. Secondly, the lack of data on anaemia in a comparable group of HIV negative children in the same setting limits our ability to interpret the prevalence results. Thirdly, failure to achieve sample size for the cohort due to limited time may have affected the results of the effect of baseline anaemia on subsequent effect on response to ART. However, the prospective cohort design, with longitudinal data collected suggests that the association between anaemia and ART response warrants further study in a larger cohort.

## Conclusions

In a group of HIV infected children in a rural Ugandan clinic the prevalence of anaemia was high but mostly mild. Microcytic-hypochromic anaemia was the most frequent type. Both the frequency and severity of anaemia were associated with advancing clinical and immunological HIV disease stage. Virological response to HAART was significantly better among children with no anaemia at baseline. Treatment with HAART improved anaemia in most HIV-infected children without additional treatments directed specifically at the anaemia. A thorough search for correctable causes of anaemia may need to be applied to HIV-infected children whose anaemia does not quickly improve on ART alone.

## Competing interests

The authors declared that they have no competing interest.

## Authors’ contributions

ENR was a recipient of Master of Medicine in Paediatrics and Child health, conceived of the study, and participated in its design and coordination, data collection, analysis and writing the manuscript. JK participated in study design and coordination, read and advised at all stages of writing the thesis and writing of the final manuscript. FB participated in the design of the study, interpretation of results and writing of the final manuscript. All authors read and approved the final manuscript.

## Pre-publication history

The pre-publication history for this paper can be accessed here:

http://www.biomedcentral.com/1471-2431/12/170/prepub
